# MONET: a database for prediction of neoantigens derived from microsatellite loci

**DOI:** 10.3389/fimmu.2024.1394593

**Published:** 2024-05-21

**Authors:** Nan Deng, Krishna M. Sinha, Eduardo Vilar

**Affiliations:** ^1^ Department of Clinical Cancer Prevention, The University of Texas MD Anderson Cancer Center, Houston, TX, United States; ^2^ Department of Gastrointestinal Medical Oncology, The University of Texas MD Anderson Cancer Center, Houston, TX, United States; ^3^ Department of Clinical Cancer Genetics Program, The University of Texas MD Anderson Cancer Center, Houston, TX, United States

**Keywords:** neoantigen, microsatellite, Lynch syndrome, mismatch repair, somatic mutation, indels

## Abstract

**Background:**

Microsatellite instability (MSI) secondary to mismatch repair (MMR) deficiency is characterized by insertions and deletions (indels) in short DNA sequences across the genome. These indels can generate neoantigens, which are ideal targets for precision immune interception. However, current neoantigen databases lack information on neoantigens arising from coding microsatellites. To address this gap, we introduce The MicrOsatellite Neoantigen Discovery Tool (MONET).

**Method:**

MONET identifies potential mutated tumor-specific neoantigens (neoAgs) by predicting frameshift mutations in coding microsatellite sequences of the human genome. Then MONET annotates these neoAgs with key features such as binding affinity, stability, expression, frequency, and potential pathogenicity using established algorithms, tools, and public databases. A user-friendly web interface (https://monet.mdanderson.org/) facilitates access to these predictions.

**Results:**

MONET predicts over 4 million and 15 million Class I and Class II potential frameshift neoAgs, respectively. Compared to existing databases, MONET demonstrates superior coverage (>85% vs. <25%) using a set of experimentally validated neoAgs.

**Conclusion:**

MONET is a freely available, user-friendly web tool that leverages publicly available resources to identify neoAgs derived from microsatellite loci. This systems biology approach empowers researchers in the field of precision immune interception.

## Introduction

Microsatellite instability (MSI) is caused by the accumulation of insertions and deletions (indels) in short-segment DNA sequences of mono-, di-, tri-nucleotide, and longer repeats known as microsatellites due to mismatch repair (MMR) deficiency. MMR deficiency is secondary to inactivating mutations in one of the four MMR genes (*MLH1, MSH2, MHS6*, and *PMS2*) or epigenetic silencing of *MLH1* (sporadic MMR deficiency) (1). These indels lead to frameshift mutations, thus resulting in the generation of mutated neoantigens (neoAgs) that are unique to tumor cells and highly unlikely to be found in normal cells. Unlike non-synonymous single-nucleotide variants (SNVs), which are mutations that typically generate neoAgs with only one altered amino acid(s), frameshift mutations typically generate completely different amino acid sequences. These frameshifted sequences have low probabilities of being tolerated by the host’s immune system. These mutated frameshift proteins possess intrinsic immunogenicity and are, therefore, attractive targets for cancer interception and therapy (2, 3).

Currently, there are several epitope databases available to facilitate *in silico* vaccine design (**Table 1**). The Immune Epitope Database (IEDB) (4) is a globally accessible gateway to experimentally validated immune epitopes, while other databases, such as AntiJen (5) and caped (6) focus on curated cancer epitopes from research manuscripts. In addition, the GNIFdb (7) and TSNAdb databases (8, 9) utilize computational approaches to predict putative neoAg based on high-frequency mutations detected in cancers. However, there is currently no antigen database dedicated to neoAgs derived from microsatellite tracts. Tumors displaying high levels of MSI (MSI-H) may harbor indels in up to 80% of microsatellite loci (10), thus suggesting that coding MSI could generate a significant number of potential neoAg candidates with high degree of immunogenicity. Leveraging this knowledge gap, we developed a new database named MicrOsatellite NEoantigen Discovery Tool (MONET) that focuses on the prediction of putative neoAgs derived from MSI in cancers.

**Table 1:** Available antigen databases.

Database	URL	Type	Target
GNIFdb	http://www.oncoimmunobank.cn/index.php	Computational	Glioma
IEDB	https://www.iedb.org/	Curated	General
TSNAdb v2.0	https://pgx.zju.edu.cn/tsnadb/	Computational	Pan cancer
dbPepNeo2.0	http://119.3.70.71/dbPepNeo2/home.html	Curated	Pan cancer
CAD v1.0	http://cad.bio-it.cn/	Curated	Pan Cancer
NeoPeptide	Not availible	Curated	Pan Cancer
NEPdb	http://nep.whu.edu.cn/.	Curated	Pan Cancer
TANTIGEN 2.0	http://projects.met-hilab.org/tadb	Curated	Pan Cancer
AntiJen	http://www.ddg-pharmfac.net/antijen/AntiJen/antijenhomepage.htm	Curated	General
caped	https://caped.icp.ucl.ac.be/	Curated	General

Here, we introduce the MONET database, which includes all possible neoAg derived from microsatellite loci present in the human reference genome. To generate MONET, we used computational algorithms to predict neoAg-derived epitopes with high affinity for CD8^+^ and CD4^+^ T cells that are specific to a variety of MHC-I and MHC-II alleles, respectively. We also evaluated the binding stability and foreignness of predicted epitopes, which are crucial factors in assessing their immunogenicity. Furthermore, we integrated data on gene expression levels of neoAgs in different tumor types leveraging the TCGA database, and mutation allele frequencies from public databases in order to provide a comprehensive immunogenicity and population coverage. Our work demonstrates that MONET has excellent coverage and outperforms other available antigen databases when tested against a set of verified epitopes derived from microsatellites. In addition, a user-friendly web-interface has been implemented and housed at https://monet.mdanderson.org/, where users can query candidate target genes and obtain curated lists of potential neoAg epitopes based on their corresponding MHC alleles without the need for complex computational efforts.

## Methods

### Generation of potential frameshift neoantigens

To generate potential frameshift neoAg derived from microsatellite loci, we utilized MSIsensor2 (https://github.com/niu-lab/msisensor2) (11) to scan the human reference genome (GRCh38) for the identification of all microsatellite loci. Short nucleotide repeats exceeding five units were identified as microsatellites. For larger repeated motifs (ranging from 2 to 5 base pairs), a minimum repeat number of three was used. Only microsatellites within protein-coding regions were retained to generate potential mutant proteins. Insertions/deletions differing by a multiple of 3 will share the same reading frame, resulting in identical downstream sequences, so that we can generate two types of downstream frameshift sequences for each identified microsatellite: 3n+1 and 3n+2 shifts of nucleotide bases, where n is an integer (-3, -2, -1, 0, 1, 2, 3, and so on). For example, in the 3n+1 series, two nucleotide deletions (n = -1 and 3n+1 = -2) in the sequence will share the same reading frame with one nucleotide insertion (n = 0, 3n+1 = 1), and four nucleotide insertions (n= 1 3n+1 = 4) and so on. This applies similarly to the 3n+2 series. Therefore, to efficiently represent these frameshift mutations, we introduce two *in silico* variants for each microsatellite: a two-nucleotide deletion (n = -1, 3n+1 = -2) and a one-nucleotide deletion (n = -1, 3n+2 = -1). These variants encompass the spectrum of frameshift mutations within each series and generate entirely distinct downstream amino acid sequences compared to the wild-type sequence, thus making them ideal neoAg candidates.

### Putative neoantigen epitopes

It is important to note the significant complexity of potential mutant amino acid sequences generated at the junction region, where the wild-type and mutant sequences meet within the microsatellite region. The size and location of indels at this junction significantly impact the resulting mutant sequence. However, based on our previous experimental data (10), very few (<5%) verified neoAg epitopes originate from these junction regions. Therefore, we excluded the sequences around the junction at which the frameshifted amino acid occurred to prevent this complexity. Then, putative neoAg epitopes were predicted towards a panel of higher frequency MHC-Class I (12) and MHC-Class II (13) alleles (**Table 2**) providing coverage for 97% and 99% of the general population, respectively. Various algorithms such as MHCflurry (14), MHCnuggets (15), NetMHC (16), PickPocket (17), SMM-align (18), NNalign (19) that are implemented in pVACtools (20) were employed to predict neoAgs. Any epitope and allele pairs with a binding affinity of IC_50_ <50 nM in any algorithm were considered potential epitopes for subsequent processes. We predicted the binding stability for Class I epitopes using NetMHCstab (21) and assessed foreignness to the human proteome using antigen.garnish (https://github.com/andrewrech/antigen.garnish) (22). Also, other characteristics that could play an important role in epitope selection such as terminal amino acids and the Gravy score (average hydropathy) were annotated.

**Table 2:** MHC Alleles used to predict putative neoantigens.

Class I Allele tested (n=27)	Class II Allele tested (n=27)
HLA-A*01:01 HLA-A*02:01 HLA-A*02:03 HLA-A*02:06 HLA-A*03:01 HLA-A*11:01 HLA-A*23:01 HLA-A*24:02 HLA-A*26:01 HLA-A*30:01 HLA-A*30:02 HLA-A*31:01 HLA-A*32:01 HLA-A*33:01 HLA-A*68:01 HLA-A*68:02 HLA-B*07:02 HLA-B*08:01 HLA-B*15:01 HLA-B*35:01 HLA-B*40:01 HLA-B*44:02 HLA-B*44:03 HLA-B*51:01 HLA-B*53:01 HLA-B*57:01 HLA-B*58:01	DRB1*01:01 DRB1*03:01 DRB1*04:01 DRB1*04:05 DRB1*07:01 DRB1*08:02 DRB1*09:01 DRB1*11:01 DRB1*12:01 DRB1*13:02 DRB1*15:01 DRB3*01:01 DRB3*02:02 DRB4*01:01 DRB5*01:01 DQA1*05:01-DQB1*02:01 DQA1*05:01-DQB1*03:01 DQA1*03:01-DQB1*03:02 DQA1*04:01-DQB1*04:02 DQA1*01:01-DQB1*05:01 DQA1*01:02-DQB1*06:02 DPA1*02:01-DPB1*01:01 DPA1*01:03-DPB1*02:01 DPA1*01:03-DPB1*04:01 DPA1*03:01-DPB1*04:02 DPA1*02:01-DPB1*05:01 DPA1*02:01-DPB1*14:01

### Annotation of neoantigens

To gain a deeper understanding of the potential epitopes, we also annotated the corresponding mutations using the Ensembl Variant Effect Predictor (VEP) (23). Annotations include associated gene names, IDs, genomic coordinates, and among others. Another critical factor for the effectiveness of neoAgs being presented by MHC is their expression level (24–26). Higher expression levels will increase the probability of these epitopes being presented by MHC molecules. We integrated the expression levels of the corresponding genes generating the neoAgs from the Cancer Genome Atlas Program (TCGA, https://www.cancer.gov/tcga) project by using all datasets that contain pairs of normal and tumor. Additionally, for the dataset with MSI status information, we distinguished and listed the MSI-H and MSS groups separately. Differentially expressed genes (Benjamini-Hochberg adjusted p-value < 0.05) between normal and cancer tissues in each of the different cancer data set were labelled. Furthermore, we annotated the frequency of the mutation and associated phenotypes using the dbSNP (RRID:SCR_002338) (27) and ClinVar (RRID:SCR_006169) database (28). This annotation provided valuable insights into the frequency and clinical implications of the mutations from where the neoAgs are derived. Moreover, we included evidence of experimentally confirmed epitopes using IEDB (RRID:SCR_006604), which contributes to the reliability and validity of the identified epitopes.

### MONET website infrastructure

We constructed the website using a microservices architecture with Docker. The backend database (MySQL) was employed to store and manage data. Express.js served as the middleware, responsible for translating HTTP requests into MySQL queries. Vue.js was utilized to develop the user interface. Nginx served as the HTTP server communicating between the containers hosting the frontend and backend components of the website.

### Comparisons of the epitope coverage derived from microsatellite loci across various databases

We selected 100 epitopes derived from mutations in microsatellite loci among the top-ranked based on their predicted immunogenicity (**Supplementary Table S2**) in LS patients and showed that 65 out of 100 predicted neoAg candidates were validated for their immunogenicity using *in vitro* ELISPOT assays (10). These peptides were used to assess the coverage across different databases. Specifically, on April 9^th^, 2024, we individually searched the 65 validated immunogenic epitopes within MONET and the 10 databases listed in **Table 1**. Then, we evaluated the number of epitopes recorded in each database. The services of dbPepNeo, NeoPeptide, and TANTIGEN databases were unavailable at the time of our research. Therefore, we reported results from MONET and the other 7 databases.

### Data Availability

Public data analyzed in this study was obtained from multiple sources including: https://www.ncbi.nlm.nih.gov/genome/, https://www.ncbi.nlm.nih.gov/snp/, https://www.ncbi.nlm.nih.gov/clinvar/, https://www.iedb.org/, https://www.cancer.gov/tcga. The epitope prediction data in this study are available at https://monet.mdanderson.org.

## Results

### Prediction of frameshift neoantigens in microsatellites

**Figure 1:**
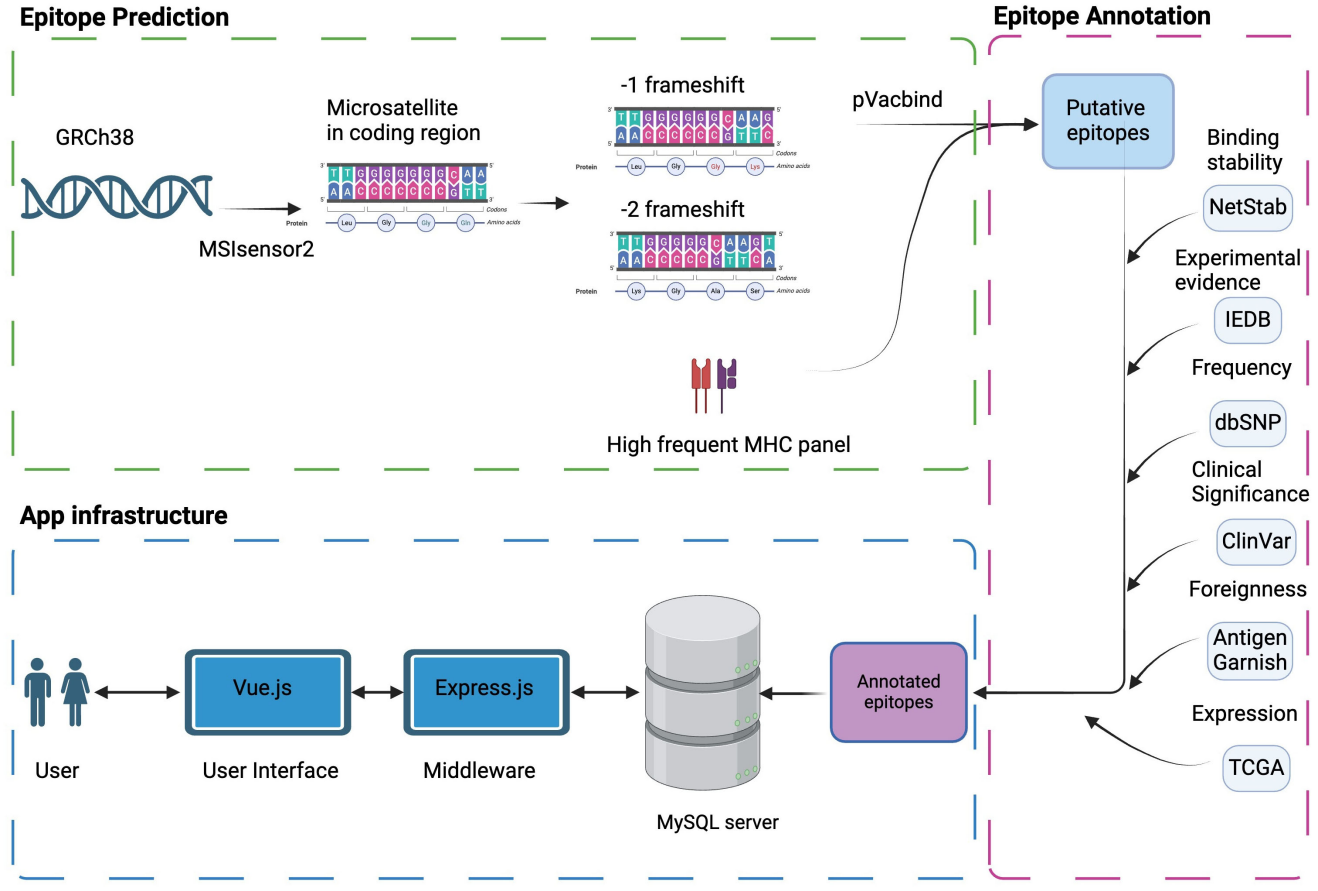
Schema of MONET. Microsatellite regions on the human reference genome GRCh38 were determined by *MSIsensor2*. The potential neoAg epitopes against high-frequency human MHC molecules were determined by *pVacbind*. The selected potential neoAgs were then annotated with other information, such as binding stability, experimental evidence, and frequency in populations, which will be useful information for vaccine design. Finally, we constructed a user-friendly interface by *Vue.js* to help users access our epitope database.

**Figure 2:**
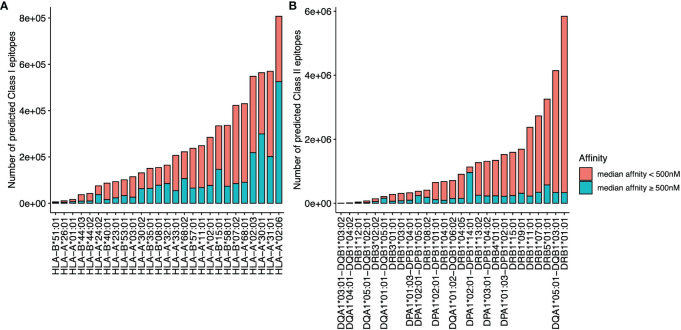
The number of predicted epitopes is restricted to different MHC I **(A)** and MHC II **(B)** alleles. The number of epitopes with a median binding affinity IC_50_<500 nM from multiple affinity binding algorithms is labeled in red, and the number of epitopes with a median affinity IC_50_ ≥500 nM is labeled in blue.

The overall data processing is depicted in **Figure 1**. We identified a total of 34,067,744 microsatellite loci in the human genome GRCh38. Among them, only 3,934,634 microsatellites were located in protein-coding regions (**Table 3**). After eliminating duplicated sequences, we obtained 492,578 unique frameshift mutation neoAg sequences (**Table 3**). From these sequences, we identified 4,449,128 MHC Class I and 15,589,846 MHC Class II potential epitopes. The number of epitopes binding to MHC Class I alleles for each allele ranged from approximately 6,000 to 800,000 (**Figure 2A**), while binders to Class II alleles displayed a higher number of predicted epitopes ranging from approximately 5,000 to 6 million (**Figure 2B**).

**Table 3:** Statistics of the MONET database.

	Count
**Microsatellites on reference genome**	34,067,744
**Microsatellites on exon regions**	3,934,634
**Genes with microsatellites on exon regions**	18,591
**Potential mutations**	11,803,902
**No duplicated downstream sequence**	492,578
**Possible Class I epitopes (best affinity <50nM)**	4,449,128
**Possible Class II epitopes (best affinity <50nM)**	15,589,846
**Mutations in dbSNP**	542,442
**Mutations in ClinVar**	13,289
**Iedb experiment verified epitope**	65,220
**Iedb experiment verified TCR and MHC information**	932
**Iedb experiment verified MHC ligand**	64,288

### Annotation of epitopes and mutations

After generation of predicted potential epitopes using our pipeline, the binding stability was annotated using antigen.garish (**Figure 1**). Then, the expression levels of the corresponding genes carrying the mutations were annotated using TCGA data. A total of 64,288 epitopes have been experimentally verified and recorded in the IEDB (**Table 3**). A total of 542,442 mutations in MONET were recorded in the dbSNP database, which contains human variants including small indels original from germline or somatic mutations. Of these mutations, 13,289 are linked to the ClinVar database, which associates human variation with their potentially clinically relevant results. While most of the variant recorded in dbSNP and ClinVar are of germline origin, 198 somatic mutations in MONET have been recorded in ClinVar. The top ClinVar phenotypes (**Table S1**) include malignant tumor of prostate (Rank 1, n = 31), carcinoma of colon (Rank 2, n= 24), and colorectal cancer (Rank 5, n=16). In one hand, these clinical records verified our putative neoAgs that might present in dMMR/MSI-H cancers. In the other hand, the limited coverage of the current clinically available databases suggests that our computational results could be very valuable in bridging this gap.

### Web interface

In MONET, the landing page can be accessed at https://monet.mdanderson.org. The key functions of MONET are: ‘Search Neoantigens’ and ‘Best Neoantigens’. Both can be accessed from the sidebar.

### Search neoantigens.

**Figure 3:**
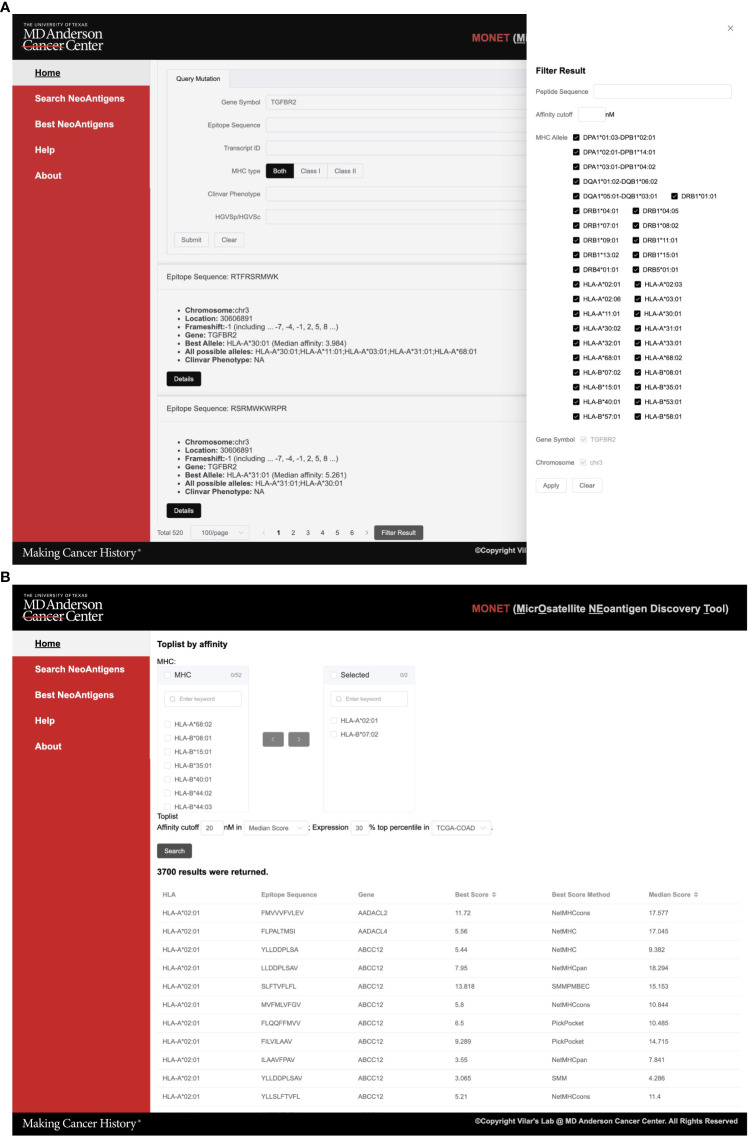
Screenshots of MONET functions. **(A)** The screenshot shows the interface for the search function of neoAgs; **(B)** The screenshot shows the interface for identifying the best neoAgs restricted to a set of MHC allele combinations.

In the ‘Search Neoantigens’ page, users have the option to search for neoAg using various parameters, including gene symbol and related IDs, epitope sequence, mutation HVGSp/HVGSc ID, and ClinVar phenotype. Moreover, users can narrow down their search by limiting it to specific MHC class I and II types. This functionality proves particularly useful when users have a specific target gene or disease in mind and would like to search into the details of a particular epitope (**Figure 3A**).

As an example, users can utilize the search function to find potential Class I neoAg epitopes resulting from mutations in *TGFBR2*. The search engine generates 520 summarized results, providing information such as peptide sequence, mutation location, related gene, and associated HLA (Human Leukocyte Antigen) alleles. To further refine the results, users can utilize the filter sidebar on the right-hand side. This allows filtering based on peptide sequence, affinity cutoff, and target HLA alleles. In cases where multiple genes are returned, users can also apply a filter based on the gene symbol. Once a specific epitope of interest is identified, users can select it to further access detailed information about the epitope (**Figure 3B**).

**Figure 4:**
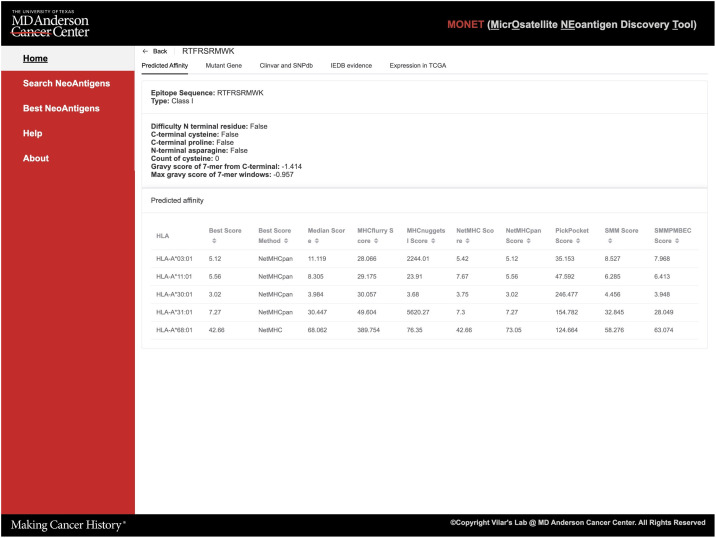
Screenshot of detailed reports of the epitope result page.

Detailed epitope information is offered in five tabs, each providing specific details (**Figure 4**): 1. *Prediction affinity* will display a table of predicted affinities generated by different algorithms for various HLA types (**Figure 4**) and other features of the epitope such as the Gravy score; 2. *The mutant gene* will show essential gene information related to the epitope, including the gene’s location, related IDs, and both wild-type and mutant sequences of the protein. The mutant sequence is highlighted in red (**Supplementary Figure S1**); 3. *ClinVar* and *dbSNP* will provide access to the gene ID and corresponding link to ClinVar and dbSNP databases. Additionally, if available, basic information about related diseases and frequency data will be presented (**Supplementary Figure S2**); 4. If the peptide has been experimentally tested in the *IEDB* (Immune Epitope Database), this tab will showcase relevant information from IEDB; 5. Users can explore the expression of the target gene in solid normal tissue versus primary solid tumors across different *TCGA databases*. Thus, this tab displays a bar plot illustrating this information (**Supplementary Figure S3**).

### Best neoantigens

Within the best neoAg search engine function, users have the capability to search for all potential epitopes specific to one or multiple HLA alleles (**Figure 3B**). This feature allows for customized filtering options, such as an affinity and expression cutoff in a specific TCGA cancer type. This functionality proves particularly valuable when users intend to identify the top potential epitopes for a specific set of HLA alleles from either an individual patient or a group of patients.

As an example, users can select HLA-A*02:01 and HLA-B*07:02 alleles along with a median affinity cutoff of 30 nM across multiple algorithms. Furthermore, they can narrow down the search to include only the top 20% most highly expressed genes in the COAD dataset (**Figure 3B**). In total, 3,186 results for HLA-A*02:01 and 2,614 results for HLA-B*07:02 returned. These potential epitopes are generated from mutations in 1,483 genes. Users have the option to download the results directly from the page, and detailed information for each peptide can be accessed on the corresponding peptide details page.

### Evaluating the coverage of neoantigens derived from microsatellite loci across various databases

**Figure 5:**
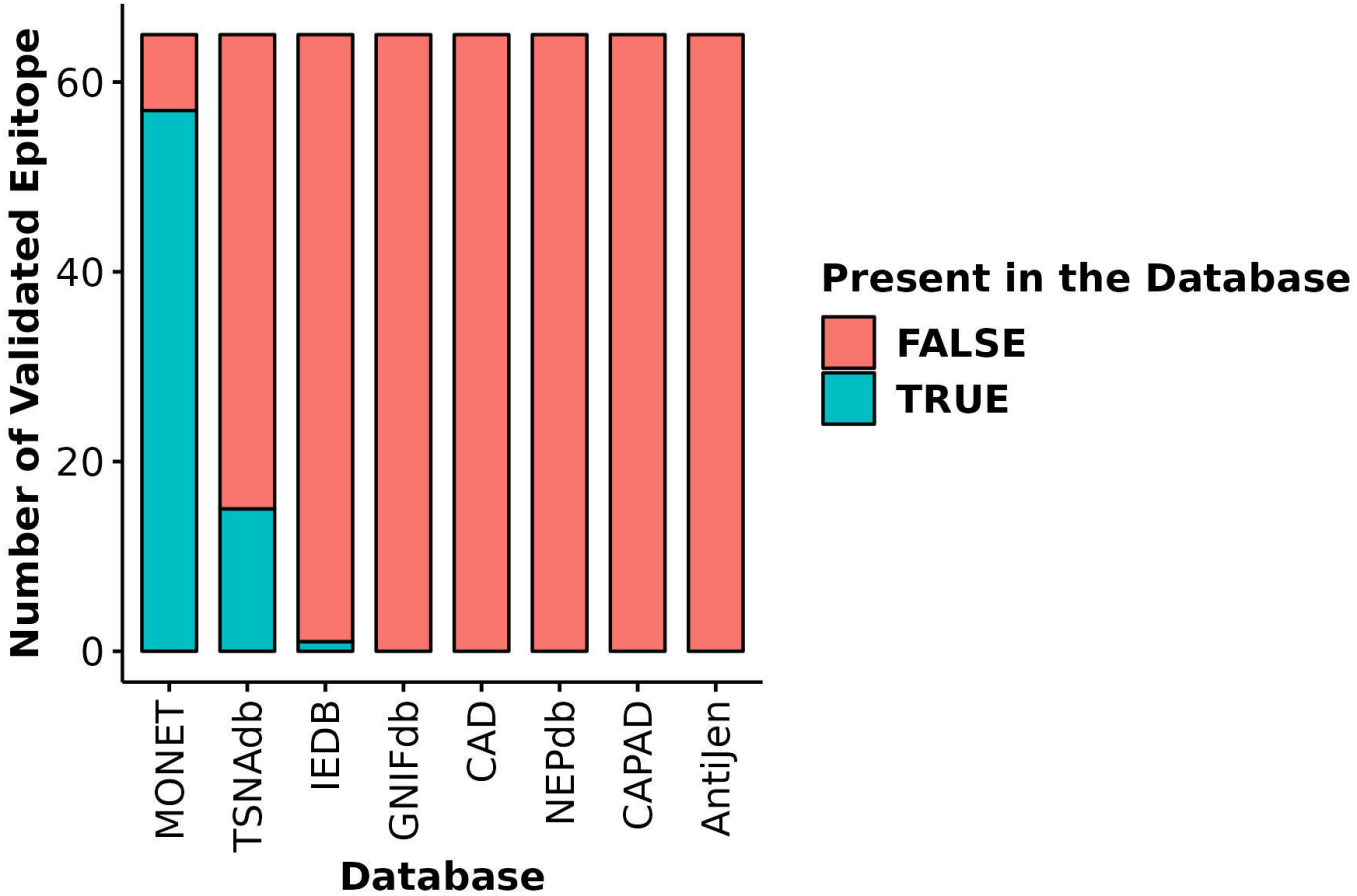
Coverage of validated epitopes derived from microsatellite loci across different databases. Y-axis: Number of validated epitopes; X-axis: Database name; Blue bars: Number of validated epitopes present in each database; Red bars: Number of validated epitopes missing from each database.

The most distinctive feature of MONET is its focus on epitopes derived from microsatellite loci that are targets of MMRd. We compared the coverage of 65 of such epitopes, the immunogenicity of which has been validated using ELISpot assays (10), across MONET and other popular databases. MONET covers 57 out of these 65 verified epitopes (**Figure 5**, **Supplementary Table S2**). In comparison to MONET, TSNAdb, only covers 15 of the 65 epitopes, and IEDB covers just one. GNIFdb, CAD, NEPdb, CAPAD, and AntiJen do not contain any of these epitopes. Therefore, MONET demonstrates excellent coverage (>85%) of our target epitopes, which are derived from microsatellite loci due to MMRd, thus significantly outperforming other available databases in this field.

## Discussion

The MONET database serves as a comprehensive resource for researchers investigating neoAg related to MSI cancers. This database covers possible neoAg derived from microsatellites within the human genome, which is specific to a panel of high-frequency MHC class I and class II alleles. Researchers can utilize the database to conduct searches based on target genes or epitope sequences, as well as target MHC alleles, to obtain comprehensive information on potential target epitopes. The user-friendly interface makes the database accessible to cancer researchers without acquiring any bioinformatic skills.

We acknowledge that MONET has several limitations. MONET solely focuses on target epitopes for humans, but we have ongoing efforts to broaden its scope and incorporate epitopes for other model systems such as mouse, rat, and rhesus, which will be particularly valuable for cancer vaccine studies using model organisms. MONET uses multiple MHC-peptide binding affinity algorithms to identify potential neoAgs. We currently treat all algorithms equally and calculate the minimum, median, or mean value of these algorithms to evaluate the peptides. However, these algorithms have large differences in performance (29, 30). In the future, we plan to exclude those algorithms with inferior performance and prioritize the weight of algorithms with the best performance based on data generated by our team or public data sets. MONET currently lacks a REST API (Representational State Transfer Application Programming Interface), which could streamline data access and analysis for bioinformaticians seeking customized or bulk queries. A REST API is planned for integration in the upcoming MONET release.

In summary, MONET is a systems biology tool that has the goal of facilitating the identification of mutated neoAgs derived from microsatellite loci by leveraging publicly available state-of-the-art tools and by providing a user-friendly online website that is freely available to the scientific community.

## Data availability statement

The original contributions presented in the study are included in the article/**Supplementary Material**. Further inquiries can be directed to the corresponding authors.

## Author contributions

ND: Conceptualization, Data curation, Formal analysis, Methodology, Software, Writing – original draft, Writing – review & editing. KS: Writing – original draft, Writing – review & editing. EV: Formal analysis, Funding acquisition, Project administration, Resources, Supervision, Writing – original draft, Writing – review & editing.
