# Supplementary material for: MONET: a database for prediction of neoantigens derived from microsatellite loci
Source: Front Immunol. 2024 May 21;15:1394593. doi: 10.3389/fimmu.2024.1394593 (PMC11148240; doi:10.3389/fimmu.2024.1394593)
Supplement: Supplementary file 1 [file DataSheet_1.docx]

**Supplementary Table**

| **ClinVar phenotype** | **Number of Somatic Variants** |
| --- | --- |
| Malignant tumor of prostate | 31 |
| Carcinoma of colon | 24 |
| Hereditary cancer-predisposing syndrome | 19 |
| Li-Fraumeni syndrome 1 | 17 |
| Colorectal cancer | 16 |
| Turcot syndrome | 14 |
| Li-fraumeni-like syndrome | 12 |
| Breast-ovarian cancer, familial, susceptibility to, 2 | 10 |
| Metastatic pancreatic neuroendocrine tumours | 10 |
| CIC-DUX Sarcoma | 9 |
| not provided | 6 |
| SLC35A2-congenital disorder of glycosylation | 6 |
| Maturity-onset diabetes of the young type 3 | 5 |
| T-cell acute lymphoblastic leukemia | 5 |
| Familial adenomatous polyposis 4 | 4 |
| Retinoblastoma | 3 |
| Tramadol response | 3 |
| Acute megakaryoblastic leukemia | 2 |
| Intellectual disability, autosomal dominant 14 | 2 |

**Table S1. Phenotypes of recorded somatic variants in ClinVar.Table S2. Coverage of validated epitopes derived from microsatellite loci across different databases.**

| **Mutant Epitope Sequence** | **MONET** | **IEDB** | **GNIFdb** | **TSNAdb** | **CAD** | **NEPdb** | **CAPAD** | **AntiJen** |
| --- | --- | --- | --- | --- | --- | --- | --- | --- |
| APRSTPRAT | TRUE | FALSE | FALSE | TRUE | FALSE | FALSE | FALSE | FALSE |
| GLMTLSKMIK | TRUE | FALSE | FALSE | FALSE | FALSE | FALSE | FALSE | FALSE |
| MTLSKMIKK | TRUE | FALSE | FALSE | FALSE | FALSE | FALSE | FALSE | FALSE |
| FLLALWECSL | TRUE | FALSE | FALSE | FALSE | FALSE | FALSE | FALSE | FALSE |
| TPQDSRQVL | TRUE | FALSE | FALSE | FALSE | FALSE | FALSE | FALSE | FALSE |
| MSDTTYKIY | TRUE | FALSE | FALSE | FALSE | FALSE | FALSE | FALSE | FALSE |
| VPVALMSAM | TRUE | FALSE | FALSE | FALSE | FALSE | FALSE | FALSE | FALSE |
| GPRMQLCTQL | TRUE | FALSE | FALSE | TRUE | FALSE | FALSE | FALSE | FALSE |
| TQLARFFPI | TRUE | FALSE | FALSE | TRUE | FALSE | FALSE | FALSE | FALSE |
| KMRKKYAQK | TRUE | FALSE | FALSE | FALSE | FALSE | FALSE | FALSE | FALSE |
| NPRRKTWKM | TRUE | FALSE | FALSE | FALSE | FALSE | FALSE | FALSE | FALSE |
| CSIPTYVKK | TRUE | FALSE | FALSE | FALSE | FALSE | FALSE | FALSE | FALSE |
| SPRTLRCL | TRUE | FALSE | FALSE | TRUE | FALSE | FALSE | FALSE | FALSE |
| LTFFHFFFFFK | TRUE | FALSE | FALSE | FALSE | FALSE | FALSE | FALSE | FALSE |
| TFFHFFFFF | TRUE | FALSE | FALSE | FALSE | FALSE | FALSE | FALSE | FALSE |
| VYQHVWLICF | TRUE | FALSE | FALSE | FALSE | FALSE | FALSE | FALSE | FALSE |
| STRSSGGTL | TRUE | FALSE | FALSE | FALSE | FALSE | FALSE | FALSE | FALSE |
| FMQFSLFSV | TRUE | FALSE | FALSE | TRUE | FALSE | FALSE | FALSE | FALSE |
| NFFMQFSLF | FALSE | FALSE | FALSE | FALSE | FALSE | FALSE | FALSE | FALSE |
| CTASASWLLR | TRUE | FALSE | FALSE | FALSE | FALSE | FALSE | FALSE | FALSE |
| KLLKKVDLK | TRUE | FALSE | FALSE | TRUE | FALSE | FALSE | FALSE | FALSE |
| KSHLGTLRK | TRUE | FALSE | FALSE | TRUE | FALSE | FALSE | FALSE | FALSE |
| SPGEARAPL | TRUE | FALSE | FALSE | FALSE | FALSE | FALSE | FALSE | FALSE |
| NWMDDSSFLLF | TRUE | FALSE | FALSE | FALSE | FALSE | FALSE | FALSE | FALSE |
| SPSSTSLAV | TRUE | FALSE | FALSE | FALSE | FALSE | FALSE | FALSE | FALSE |
| AMAQVTHPL | TRUE | FALSE | FALSE | FALSE | FALSE | FALSE | FALSE | FALSE |
| GPAGLPLAM | TRUE | FALSE | FALSE | FALSE | FALSE | FALSE | FALSE | FALSE |
| SLMEQIPHL | TRUE | TRUE | FALSE | TRUE | FALSE | FALSE | FALSE | FALSE |
| FLVSVIFFV | FALSE | FALSE | FALSE | FALSE | FALSE | FALSE | FALSE | FALSE |
| GMKSSVRLK | TRUE | FALSE | FALSE | TRUE | FALSE | FALSE | FALSE | FALSE |
| VILTGTPQV | FALSE | FALSE | FALSE | TRUE | FALSE | FALSE | FALSE | FALSE |
| VYSGNLLSF | TRUE | FALSE | FALSE | TRUE | FALSE | FALSE | FALSE | FALSE |
| LSSVSFFLY | FALSE | FALSE | FALSE | FALSE | FALSE | FALSE | FALSE | FALSE |
| KLTETVYST | FALSE | FALSE | FALSE | FALSE | FALSE | FALSE | FALSE | FALSE |
| CPLLGKVAW | TRUE | FALSE | FALSE | FALSE | FALSE | FALSE | FALSE | FALSE |
| SLSLLPQFPL | TRUE | FALSE | FALSE | FALSE | FALSE | FALSE | FALSE | FALSE |
| SLSQEQAWPK | TRUE | FALSE | FALSE | FALSE | FALSE | FALSE | FALSE | FALSE |
| RWIPACLSLI | TRUE | FALSE | FALSE | FALSE | FALSE | FALSE | FALSE | FALSE |
| SVFQMTFIK | TRUE | FALSE | FALSE | FALSE | FALSE | FALSE | FALSE | FALSE |
| RVWSFLFLR | TRUE | FALSE | FALSE | FALSE | FALSE | FALSE | FALSE | FALSE |
| SIYTGLSRK | TRUE | FALSE | FALSE | FALSE | FALSE | FALSE | FALSE | FALSE |
| RTNRLFGMK | TRUE | FALSE | FALSE | FALSE | FALSE | FALSE | FALSE | FALSE |
| ESDNLGLLY | TRUE | FALSE | FALSE | FALSE | FALSE | FALSE | FALSE | FALSE |
| MPAPRAITI | TRUE | FALSE | FALSE | TRUE | FALSE | FALSE | FALSE | FALSE |
| RVFILLLSDK | TRUE | FALSE | FALSE | FALSE | FALSE | FALSE | FALSE | FALSE |
| VPMMLTILIW | TRUE | FALSE | FALSE | FALSE | FALSE | FALSE | FALSE | FALSE |
| KYIQVKKLI | TRUE | FALSE | FALSE | FALSE | FALSE | FALSE | FALSE | FALSE |
| KTGCRGLLGK | FALSE | FALSE | FALSE | FALSE | FALSE | FALSE | FALSE | FALSE |
| WPASRLIFW | TRUE | FALSE | FALSE | FALSE | FALSE | FALSE | FALSE | FALSE |
| MPSMTDTQM | TRUE | FALSE | FALSE | FALSE | FALSE | FALSE | FALSE | FALSE |
| VWQITGLKF | TRUE | FALSE | FALSE | FALSE | FALSE | FALSE | FALSE | FALSE |
| YYICMKERTL | TRUE | FALSE | FALSE | FALSE | FALSE | FALSE | FALSE | FALSE |
| RYMKYMLWMF | TRUE | FALSE | FALSE | FALSE | FALSE | FALSE | FALSE | FALSE |
| SYPSSLSVF | TRUE | FALSE | FALSE | FALSE | FALSE | FALSE | FALSE | FALSE |
| SYLNILPALF | TRUE | FALSE | FALSE | FALSE | FALSE | FALSE | FALSE | FALSE |
| LFMNVQFLF | TRUE | FALSE | FALSE | FALSE | FALSE | FALSE | FALSE | FALSE |
| SYAKISPLW | FALSE | FALSE | FALSE | FALSE | FALSE | FALSE | FALSE | FALSE |
| QTDILHFLF | TRUE | FALSE | FALSE | FALSE | FALSE | FALSE | FALSE | FALSE |
| RYPSLLEQQI | TRUE | FALSE | FALSE | FALSE | FALSE | FALSE | FALSE | FALSE |
| KWAGLQLPF | TRUE | FALSE | FALSE | FALSE | FALSE | FALSE | FALSE | FALSE |
| FFMATSSHRF | FALSE | FALSE | FALSE | TRUE | FALSE | FALSE | FALSE | FALSE |
| SYRWLSLFDF | TRUE | FALSE | FALSE | FALSE | FALSE | FALSE | FALSE | FALSE |
| RYPSFPLRL | TRUE | FALSE | FALSE | TRUE | FALSE | FALSE | FALSE | FALSE |
| LSDANQMLF | TRUE | FALSE | FALSE | TRUE | FALSE | FALSE | FALSE | FALSE |
| QTDDHMEIFY | TRUE | FALSE | FALSE | FALSE | FALSE | FALSE | FALSE | FALSE |

**Supplementary Figure Legends.**

**Figure S1**. Screenshot of the mutant gene tab of the epitope page showing detailed gene mutation location and protein sequences of the wild-type and mutant alleles.

**Figure S2**. Screenshot of the ClinVar and SNPdb tab of the epitope page showing potential clinical evidence and frequency of the mutation curated from ClinVar and SNPdb databases.

**Figure S3**. Screenshot of the TCGA expression tab showing the expression of the corresponding gene in the TCGA database. Each column shows a different dataset in TCGA, and the green bars represent the expression in normal tissues, while the red bars represent the expression in primary tumors.


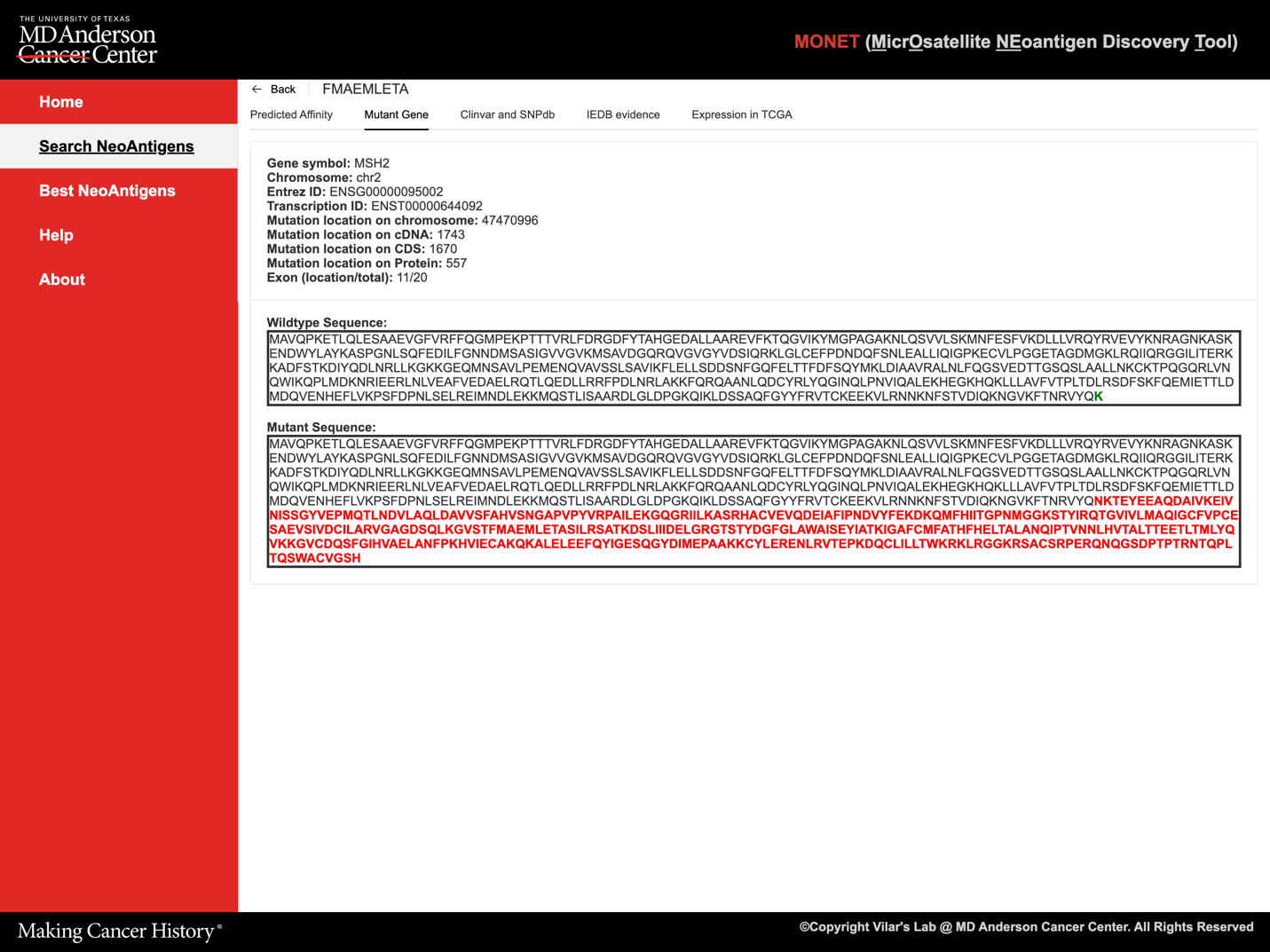


**Figure S1**. Screenshot of the mutant gene tab of the epitope page showing detailed gene mutation location and protein sequences of the wild-type and mutant alleles.


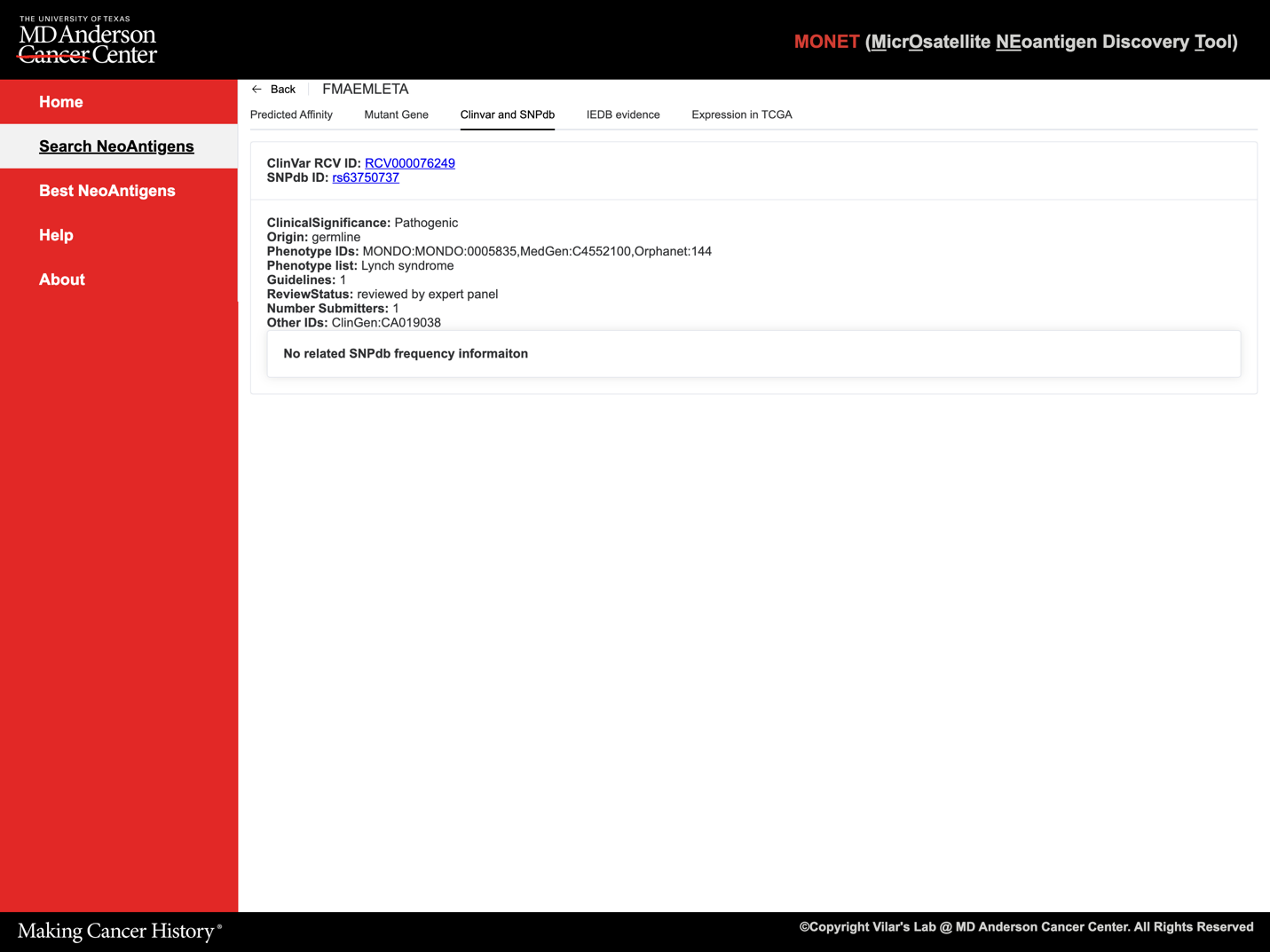
 **Figure S2**. Screenshot of the ClinVar and SNPdb tab of the epitope page showing potential clinical evidence and frequency of the mutation curated from ClinVar and SNPdb databases.


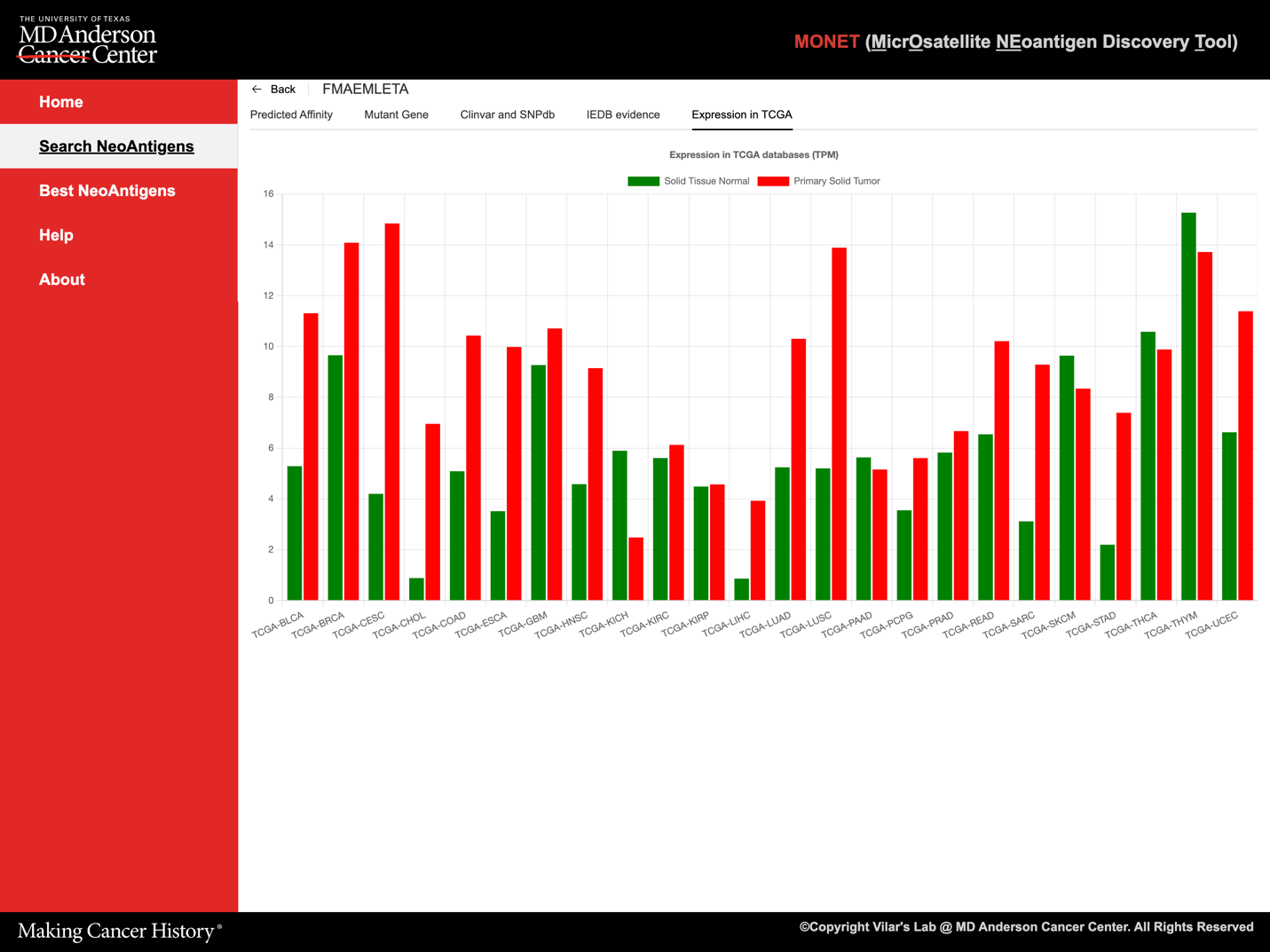


**Figure S3**. Screenshot of the TCGA expression tab showing the expression of the corresponding gene in the TCGA database. Each column shows a different dataset in TCGA, and the green bars represent the expression in normal tissues, while the red bars represent the expression in primary tumors.
